# Supplementary material for: Patterns of Intron Gain and Loss in Fungi
Source: PLoS Biol. 2004 Nov 30;2(12):e422. doi: 10.1371/journal.pbio.0020422 (PMC532390; doi:10.1371/journal.pbio.0020422)
Supplement: Table S1 — Also available at http://genes.mit.edu/NielsenEtAl/. (4.3 MB ZIP). [file pbio.0020422.st001.zip › NielsenEtAl/html/601.html]

AN7709.1.NCU03730.1.MG08167.1.FG05131.1


```
 CLUSTAL W (1.82) Multiple Sequence Alignments - Introns Inserted


Sequence 1: NCU03730.1	475 aa
Sequence 2: MG08167.1	497 aa
Sequence 3: FG05131.1	453 aa
Sequence 4: AN7709.1	465 aa
Alignment Length: 516 aa
Number Identitical Residues: 127 aa
Alignment Score (without introns) 7349


MG08167.1 	MANLS-------EKDLIFSEQANHNFSRILGALKRSNLSIENRLRSIRDDADFVASVHAA
NCU03730.1	MPAPT-------LADIVFSEQANHNFSKILGDLKKSNLSITNRLKSIQHDAAFVHEVADV
FG05131.1 	MPDPH-------FSDLILPQNEQLSMSQLMASLKRTTLTIHNRLTSIQTDAEFVDNVSHS
AN7709.1  	MGSVANSDFPVSVSALHFPSSEQLSVSQTLASLRRSALSVNNRLRSIEADAAFVREVADH
          	*    .:. . :   : :... : ..*: :. *::: *:: *** **. ** ** .*   

MG08167.1 	YATGPNSRRPLVANERCGSWYVPPGSKGGSAYFKSTDGHQGQWRFSTRRLNLHLLPLIGE
NCU03730.1	LG------LPLVANERCGSWYIDPQLKRSSAYFKSTDGHTGQWKFSTRRLNFHLLEVIGR
FG05131.1 	YAP-----RPLVANERCGSWYIPPERKGASAYFKSTDGHERAWKFSTRRLNLHLVEIIEK
AN7709.1  	YG------LPLIANERCGSWYIPPDVKSGSAYFKSTDGHTGQWDFSFRRLNLQILPIARH
          	 .       **:*********: *  * .**********   * ** ****:::: :  .

MG08167.1 	HDG2CIIVDSTRRGKR~IPDALSKTIPIWCCVVNRVLFPNLPAHAHDVFVPPYAVSDSEC
NCU03730.1	NDG2CIIVDSTRRGKR1MPDALSKTIPIWCTVLNQALFPSHP-ESHTLHVPPTVVSSSEH
FG05131.1 	NDG2IIIVDSTRRGKR1MPDALSSTIPIWCTVLNLAIIPSHP-SSSKLYLPSHLPATT-H
AN7709.1  	HGG2CIIVDSTRRGKL1MPDALSKTIPIWCAVFNRALFPSET-AYHSVELPPNYLGASEE
          	:.*  **********  :*****.****** *.* .::*. .     : :*.   . :. 

MG08167.1 	AQMLSRIPEHVASFQ~RLDPDLASLRSQIRKPLRPMWVIQPESGAATAELPGFDNDDSND
NCU03730.1	SQMSSRLPFFLSSLL~ALQPDIPSLRQNIQKPLRPLWVTPDD--ELTPEM----------
FG05131.1 	SQISTLIPGFVASLK~ALNLNLP---TCLTKPLRPFWVTPDS-TLPSPED----------
AN7709.1  	SQIEQRIDGFVHSLK0SLKLDLDDLKQQLGKPIRIAWANRTYFHPTDLSK----------
          	:*:   :  .: *:   *. :: .    : **:*  *.          .           

MG08167.1 	GQATPATVIFEDFHPVICCTSSRRVVGTEMSESGYIQGAGDDTENWAHGLTPPVFWAHAD
NCU03730.1	-----VDEIKVSFHPVVCCTSSRRVVGTEMSEEGYIQGAGDDTENWAHGLTAPLFWANQR
FG05131.1 	-----TESIFEDFRPVICCTASRRVVGSEMDEGGYIQGAGDDTEMWAHGLEAPLFWENVD
AN7709.1  	---------GEAYNLFVLCSASKRVHGAEISEGGYIQGAGDDSEAWAHGLTPAVFWAHKS
          	            :. .: *::*:** *:*:.* *********:* ***** ..:** :  

MG08167.1 	RLLSTPEADLPDLIRSLVAESAAGSAQAGTAAAQELTSFLAVAALPLPSTTTEQAGPASP
NCU03730.1	QLLETPEGDLPGLIEMLVANSEAGGQAVG--ELKRVAPRLLVGALSAEEKALTSSSQEN-
FG05131.1 	ELLETSEAELPDLISRIVDEQATSKKDEG--FQLQIVPHISVCALPLPAMPAECR-----
AN7709.1  	TLLTRAEEDLPEVIADLMQESRKQGSGQQ---ATLIAPSQNLYVSRTDPSLAANG-----
          	 **  .* :** :*  :: :.              :..   : .                

MG08167.1 	TAVVQLSPVVTKPETWHKSPTLIDVGIGKGKLASRNLRQALPAICSFVR---EAALKQQQ
NCU03730.1	ICVVSLVPKTTEKETWEKSKRHMEVGLGKSKTASRFLREALPSICDFVSRFLQECADADA
FG05131.1 	---IAITQDTTPKDSWLKSKTYMQAGLGKSKLASRNLRTALPEICDFAAKYLASVEEPGE
AN7709.1  	-LYDLVIDCNSSAETTEEKPKRLNLGCISSKQGGRDLRNHLDKVRDFVN--LHLASSPSQ
          	     :    :  ::  :.   :: *  ..* ..* **  *  : .*.        .   

MG08167.1 	QKKTAEG----TKILVACESGKDVSVGVALALACRCLDEDGNVLPADQAQVERRPVTK~D
NCU03730.1	ETGTSTGKDANKEIVLLCESGRDLSVGVALALYCWCFVDSAGTVRSAADQGQNH--NK~A
FG05131.1 	Q-----------RIAVACESGKDLSVGVALAISCYLFGDEGKVR----DSTKNVSFTK~N
AN7709.1  	S------------LLVMCESGKDLSAGTLLTIICLFYNDAGKLNR--YQHYSNLTLTV0Y
          	.            : : ****:*:*.*. *:: *    : .          .. . .   

MG08167.1 	FIRVRLGRIMTAMPEANPSRATLQSVNSFLMDWRG-----
NCU03730.1	SIRVKLGHIMTAFPDANPSRSTLQSVNSFLMDWRN-----
FG05131.1 	LIKSRLGLIMTAYPGANPSRATLQSVNSFLMDWRK-----
AN7709.1  	WTNDLLILNSLHNGSSTSLVQSVQHRNILIGDVEVEYVGI
          	  .  *         :..   ::*  * :: * . .  .
```
